# TCF21 hypermethylation in genetically quiescent clear cell sarcoma of the kidney

**DOI:** 10.18632/oncotarget.4682

**Published:** 2015-06-28

**Authors:** Saskia L. Gooskens, Samantha Gadd, Jaime M. Guidry Auvil, Daniela S. Gerhard, Javed Khan, Rajesh Patidar, Daoud Meerzaman, Qing-Rong Chen, Chih Hao Hsu, Chunhua Yan, Cu Nguyen, Ying Hu, Charles G. Mullighan, Jing Ma, Lawrence J. Jennings, Ronald R. de Krijger, Marry M. van den Heuvel-Eibrink, Malcolm A. Smith, Nicole Ross, Julie M. Gastier-Foster, Elizabeth J. Perlman

**Affiliations:** ^1^ Department of Pediatric Hematology and Oncology, Erasmus MC - Sophia Children's Hospital, Rotterdam, The Netherlands; ^2^ Department of Pediatric Oncology, Princess Máxima Center for Pediatric Oncology, Utrecht, The Netherlands; ^3^ Department of Pathology, Ann and Robert H. Lurie Children's Hospital of Chicago, Northwestern University's Feinberg School of Medicine and Robert H. Lurie Cancer Center, Chicago, IL, USA; ^4^ Office of Cancer Genomics, National Cancer Institute, Bethesda, MD, USA; ^5^ Genetics Branch, Oncogenomics section, National Cancer Institute, Bethesda, MD, USA; ^6^ Computational Genomics Research Group, Center for Biomedical Informatics and Information Technology, National Cancer Institute, National Institutes of Health, Bethesda, MD, USA; ^7^ Department of Pathology, St. Jude Children's Research Hospital, Memphis, TN, USA; ^8^ Department of Pathology, Josephine Nefkens Institute, Erasmus MC, Rotterdam, The Netherlands; ^9^ Department of Pathology, Reinier de Graaf Hospital, Delft, The Netherlands; ^10^ Cancer Therapy Evaluation Program, National Cancer Institute, Bethesda, MD, USA; ^11^ Department of Pathology and Laboratory Medicine, Nationwide Children's Hospital, Ohio State University College of Medicine, Columbus, OH, USA

**Keywords:** clear cell sarcoma of the kidney, whole genome sequencing, methylation, TCF21, TARID

## Abstract

Clear Cell Sarcoma of the Kidney (CCSK) is a rare childhood tumor whose molecular pathogenesis remains poorly understood. We analyzed a discovery set of 13 CCSKs for changes in chromosome copy number, mutations, rearrangements, global gene expression and global DNA methylation. No recurrent segmental chromosomal copy number changes or somatic variants (single nucleotide or small insertion/deletion) were identified. One tumor with t(10;17)(q22;p13) involving fusion of *YHWAE* with *NUTM2B* was identified. Integrated analysis of expression and methylation data identified promoter hypermethylation and low expression of the tumor suppressor gene *TCF21* (*Pod-1/capsulin/epicardin)* in all CCSKs except the case with t(10;17)(q22;p13). *TARID*, the long noncoding RNA responsible for demethylating *TCF21*, was virtually undetectable in most CCSKs. *TCF21* hypermethylation and decreased *TARID* expression were validated in an independent set of CCSK tumor samples. The presence of significant hypermethylation of *TCF21,* a transcription factor known to be active early in renal development, supports the hypothesis that hypermethylation of *TCF21* and/or decreased *TARID* expression lies within the pathogenic pathway of most CCSKs. Future studies are needed to functionally verify a tumorigenic role of *TCF21* down-regulation and to tie this to the unique gene expression pattern of CCSK.

## INTRODUCTION

Clear Cell Sarcoma of the Kidney (CCSK) comprises approximately 5% of all renal malignancies in children, and is observed most often below 3 years of age [1, 2]. CCSK exhibits considerable morphologic diversity, which often makes accurate pathologic diagnosis difficult [2, 3]. The current intensive treatment schedules for CCSK, including high doses of anthracyclines and radiotherapy, have resulted in a significant improvement in the outcome of these patients with 5-year event-free and overall survivals of approximately 80% and 90%, respectively [2, 4]. However, especially in patients with advanced-stage disease and following relapse, outcome is unsatisfactory. In addition, the required intensive therapy may result in serious toxicity [5, 6] and treatment options for patients with relapsed CCSK are limited [7, 8]. Therefore, prognostic markers that may predict tumor behavior and new molecular targets for therapy are needed.

Over the last two decades, three independent case reports have documented a clonal balanced translocation involving t(10;17)(q22;p13) in CCSK tumor samples [9-11]. This rearrangement involves genomic breakpoints in *YWHAE* intron 5 (17p13) and *NUTM2* intron 1 (10q22), resulting in fusion of *YWHAE* exon 5 to *NUTM2* exon 2 (*NUTM2* is also known as *FAM22*) [12, 13]. In a larger study, this transcribed chimeric transcript was identified in 6/50 CCSKs [12]. Of note, the same t(10;17)(q22;p13) translocation, involving the same introns, has been identified to be recurrent in high-grade endometrial stromal sarcomas [13]. Another fusion transcript involving *IRX2* and *TERT*, caused by an interstitial deletion of 5p, was recently reported in a single tumor classified as CCSK [14]. The observation that CCSK tissue sections stained strongly with EGFR antibodies led to the identification of gene amplification of *EGFR* in 1/12 cases and somatic *EGFR* mutation in 1/12 cases, with both samples additionally harboring somatic mutations in *PTEN* [15]. Lastly, despite the heterogeneity of the above outlined genetic changes, gene expression analysis demonstrates strong activation of genes of the Sonic Hedgehog signaling pathway and Akt-driven cell proliferation pathway in all CCSKs [16]. The current report describes the results of the first comprehensive molecular characterization of 13 CCSKs.

## RESULTS

### Chromosome segment copy number analysis

**Figure 1:**
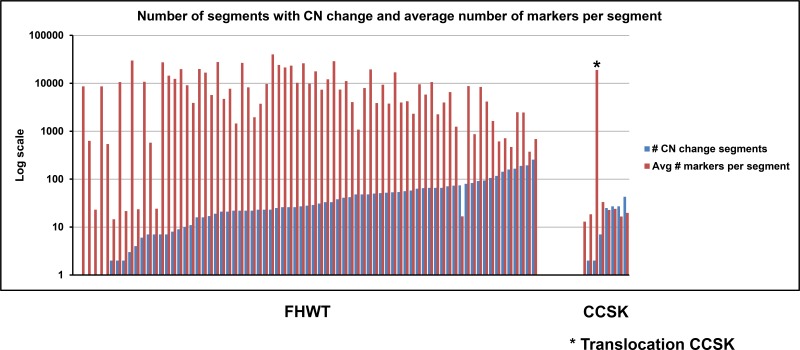
Number of segments with copy number change and average number of markers per segment Distribution of number of segments defined by > 8 markers with log2 ratios of < −0.5 or > +0.5 in 76 favorable histology Wilms tumors (FHWT) and 13 discovery set CCSKs is illustrated by the blue bars (1 FHWT and 5 CCSKs contained no gains or losses, therefore no bars are visible). The red bars illustrate the average number of markers per segment. The asterisk indicates the single CCSK of the discovery set containing the t(10;17)(q22;p13). This tumor had loss of 17p and gain of 10q resulting in a large number of segments identified from these regions of copy number change.

SNP arrays and relative coverage generated by whole genome sequencing were used to analyze chromosome segment copy number gain and loss in the 13 paired normal and CCSK tumor samples of the discovery set. The majority of CCSKs demonstrated a very small number of segmental areas of chromosomal gain or loss, and no recurrent copy number gains or losses were identified. In particular, there was no evidence of amplification of the *EGFR* locus. Segments of gain or loss of autosomal chromosomes containing at least 8 markers and showing log2 ratios < −0.5 or > +0.5 are shown in Figure 1, compared with 76 favorable histology Wilms tumors. In addition to showing fewer segments of copy number change, the average length of each segment (as measured by the number of markers per segment) was small in CCSKs in comparison with favorable histology Wilms tumors, which often shown gain or loss of entire chromosomal arms. In one CCSK, gain of distal 10q22-qter and loss of proximal 17p13-pter was identified, which will be shown below to harbor a t(10;17)(q22;p13) translocation.

### Identification of somatic mutations in CCSK

Paired CCSK tumor and normal kidney or peripheral blood samples of the 13 patients in the discovery set were sequenced at an average coverage of 59 (range 54-63). There were a total of 41 variants with somatic score ≥ −10, somatic rank ≥ 0.1 and Fisher's Exact Test (FET) score ≥ 13; RNA-sequencing found 5/41 variants to be expressed (total coverage ≥ 10, variant allelic fraction ≥ 0.2) (Supplemental Table 1). None had a MutSig p-value of < 0.05. Of the 5 verified variants, one was a known polymorphism (TOP3B) and one was not predicted to be damaging by PolyPhen2 (*PJA2*); the 3 remaining variants (*CAMSAP1, DHTKD1, KIF26A*) involved genes present in COSMIC (version 69) [17], and were predicted to be damaging by PolyPhen2 [18]. *KIF26A* functions within the Akt pathway as a suppressor of *GRB2* [19], *CAMSAP1* is a microtubule-binding protein that plays a role in cytoskeletal organization [20], and *DHTKD1* encodes a component of a mitochondrial 2-oxoglutarate-dehydrogenase-complex-like protein involved in the degradation pathways of several amino acids [21].

We then searched for germline variants within the exons of the 27,829 genes in COSMIC version 69 that were not present in dbSNP (Build 138) (unless the specific variant was also present in COSMIC) and that were predicted to be damaging by Polyphen2. None of the recurrent variants had a MutSig p-value < 0.05 or had clear functional relevance (Supplemental Table 1). This is in keeping with the absence of reports of either familial or bilateral CCSKs in the literature.

RNA sequencing data of the 13 CCSKs in the discovery set were additionally analyzed for fusion transcripts using two different computational methods, deFuse and TopHat-Fusion. Only one consistent fusion transcript was identified: this involved intron 5 of *YWHAE* on chromosome 17 and intron 1 of *NUTM2B* on chromosome 10 and was found in the sample noted above to have gain of distal 10q22-qter and loss of proximal 17p13-pter. This fusion transcript was verified by RT-PCR. Of note, while the majority of high grade endometrial stromal sarcomas contained a balanced t(10;17)(q22;p13), one endometrial stromal sarcoma has similarly been reported with deletion of the derivative chromosome containing distal 17p [13], supporting the suggestion that the remaining derivative chromosome is responsible for biologic activity.

### Gene expression analysis

**Figure 2:**
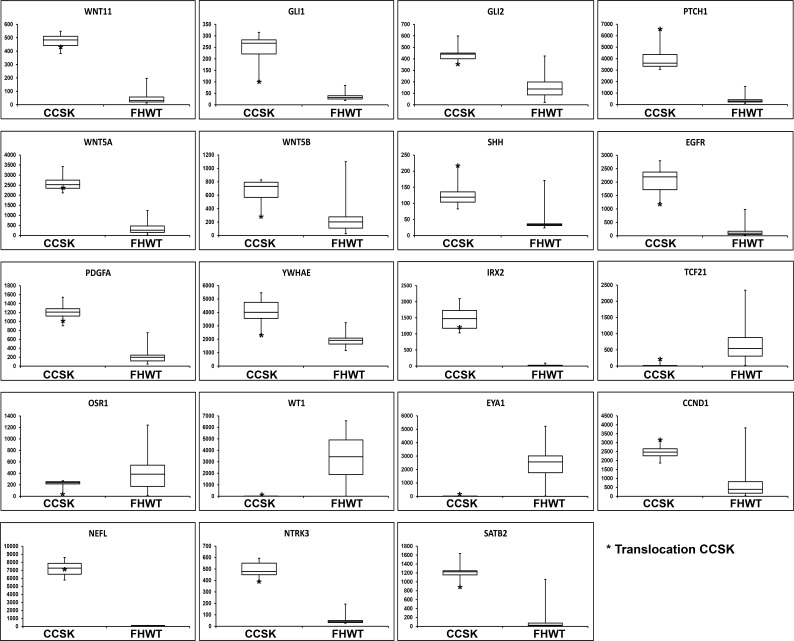
Box plots of genes of interest differentially expressed in CCSK Each box plot compares the expression of 13 CCSKs of the discovery set (left side) with 76 favorable histology Wilms tumors (FHWT) (right side). The y-axis represents the normalized expression value. The bottom and top of the box represent the first and third quartiles, respectively, the band inside the box represents the median, and the whiskers represent the maximum and minimum values. Expression of the CCSK of the discovery set containing the t(10;17)(q22;p13) is marked by an asterisk.

**Figure 3:**
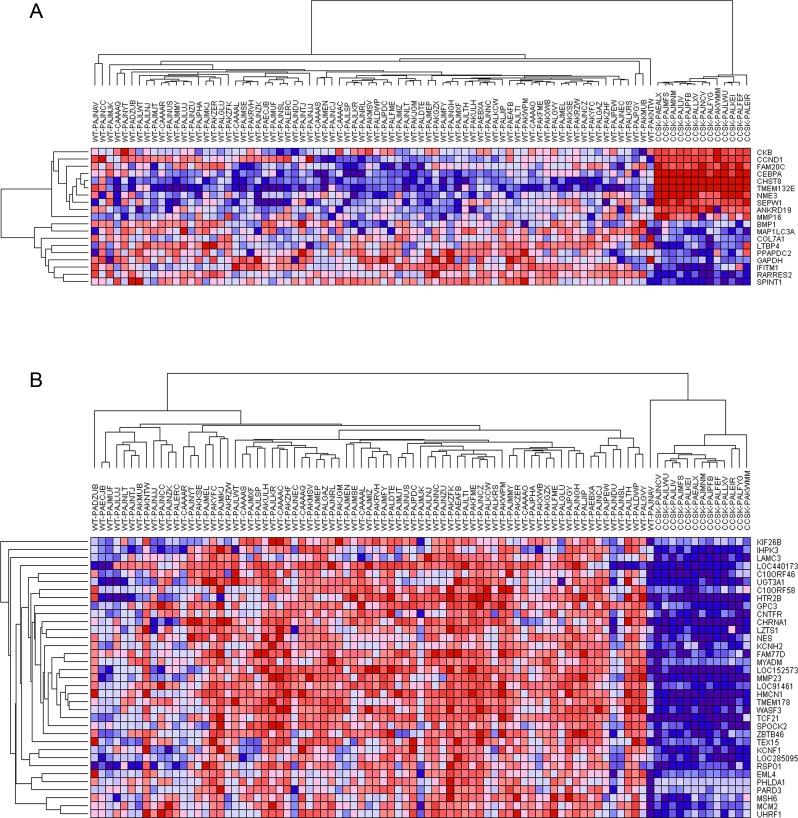
Hierarchical analysis of gene expression Genes under-expressed are shown in blue, and those over-expressed are shown in red. CCSK-PAKWMM is the CCSK containing the t(10;17)(q22;p13). **A.** Genes associated with t(10;17)(q22;p13). Those genes reported to be differentially expressed in t(10;17)(q22;p13)-bearing endometrial stromal sarcomas compared to endometrial stromal sarcomas without the translocation were entered into GSEA and demonstrated to be significantly enriched in all CCSKs (see text). Hierarchical analysis of those genes ranked highest by GSEA for their ability to distinguish CCSKs from favorable histology Wilms tumors (FHWT) is illustrated. **B.** Genes associated with *TCF21* expression. Those genes with a Pearson correlation coefficient (PCC) of ≥ 5.0 or ≤ −5.0 when comparing *TCF21* and all remaining genes on the Affymetrix U133+2.0 chip within 76 favorable histology Wilms tumors (WT) were identified. The expression of these genes within both 76 favorable histology Wilms tumors and 13 CCSK samples was then entered into GSEA and only those with a positive PCC were significantly enriched in CCSKs (see text). Hierarchical analysis of those genes ranked highest by GSEA for their ability to distinguish CCSKs from favorable histology Wilms tumors is illustrated.

Gene Set Enrichment Analysis (GSEA) comparing the 13 CCSKs within the discovery set to 76 favorable histology Wilms tumor TARGET samples demonstrated significant enrichment of two gene sets (KEGG_HEDGEHOG_SIGNALING_PATHWAY, KEGG_BASAL_CELL_CARCINOMA). These had in common over-expression of genes involved in the Sonic Hedgehog pathway including *WNT11, GLI1, GLI2, WNT5A, WNT5B, PTCH1,* and *SHH* (Figure 2), confirming our prior results from a different comparison set [16]. Genes significantly differentially expressed by SAM (n = 5,490) are provided through the TARGET Data Matrix. Of particular note, increased expression was identified for both *EGFR* (fold change 35.10, p 6.07E-36) and *PDGFA* (fold change 6.86, p 1.51E-36) (Figure 2). Low to no expression of markers of the intermediate mesoderm (including *OSR1, EYA1, WT1*) was identified in CCSK, whereas genes involved in neural development were over-expressed (including *NEFL, NTRK3, IRX2* and *SATB2*) (Figure 2). *YWHAE* was significantly up-regulated in all CCSKs (Figure 2), while there was no difference in the expression of *NUTM2* when comparing all CCSKs with favorable histology Wilms tumors. The previously reported endometrial stromal sarcomas containing the t(10;17)(q22;p13) were shown to have a distinctive expression profile compared to endometrial stromal sarcomas without the translocation [13]. We therefore analyzed those genes differentially up- and down-regulated (n = 16 and n = 32, respectively) in translocation-bearing versus non-bearing endometrial stromal sarcoma using GSEA, and both were significantly enriched in all CCSKs, regardless of translocation status, compared with Wilms tumor (FDR < 0.01 and 0.08 for genes up- and down-regulated in ESS, respectively). This includes *CCND1* (Figure 2), which has also been reported to be over-expressed by immunohistochemistry in CCSKs [22]. Hierarchical clustering of those genes differentially expressed in endometrial stromal sarcoma and ranked highest by GSEA for their ability to distinguish CCSKs from favorable histology Wilms tumors from both gene lists, is illustrated in Figure 3A.

To identify pathways and processes involved in the progression of CCSK, primary samples of CCSKs that did not subsequently relapse (n = 7) were compared with the primary samples of tumors that eventually relapsed (n = 6); no significant difference in gene expression was identified (p 0.3178).

### Methylation analysis

To identify if differences in DNA methylation may be implicated in CCSK tumorigenesis, an integrative approach was used. Global methylation analysis using Illumina 450KBeadChips was performed on 11/13 discovery samples for which sufficient DNA was available. Probes with a significant correlation (p < 0.05, both negative and positive) between expression and methylation were identified (n = 483). Genes containing at least 5 probes with both average ß-values of > 0.75 or < 0.25 and a standard deviation of < 0.25 were selected for further analysis. The resulting eight genes (*EMX2, HOXA1, IRX4, MCF2L, OSR2, PAX2, SOX1* and *TCF21*) are provided in Supplemental Table 2, annotated using GRCh37 to provide the location of the probe within the gene and the relationship of the probe within a CpG island. In addition, Supplemental Table 2 provides whether or not the promoter of the gene was known to be within a DNA methylation valley (DMV), as described by Xie *et al.* [23] (DMVs are long regions of hypomethylation identified in genes that drive early development). Analysis of these data yielded the following observations: (1) hypermethylation of *HOXA1* and *OSR2*, and hypomethylation of *MCF2L* was dispersed rather than localized, interspersed with probes showing a normal methylation pattern, and the differentially methylated probes were not located in regions that regulate transcription and (2) *PAX2, SOX1, IRX4* and *EMX2* displayed hypomethylation in both CCSKs and favorable histology Wilms tumors in regions located within or downstream of DMVs [23]; coordinated methylation patterns were not apparent by Integrative Genome Viewer (IGV).

**Figure 4:**
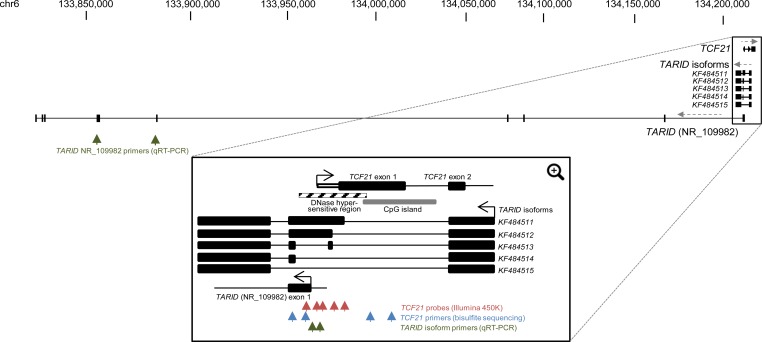
*TCF21* and *TARID* genomic regions Schematic view of the full UCSC depiction of the genomic regions of *TCF21* and *TARID* (NR_109982) (Top). Black boxes indicate coding exons and direction of transcript strands is indicated by grey dashed arrows. The green arrows indicate the sites of the location of the primers used to detect expression of *TARID* (NR_109982) (spanning exons 5 and 6). Below, a magnified region including the first two exons of *TCF21* and the *TARID* isoforms as reported by Arab *et al* [26] is shown. The arrows indicate the sites of the probes analyzed by 450K Illumina analysis for *TCF21* methylation (red), bisulfite sequencing for *TCF21* methylation (blue), and qRT-PCR for *TARID* isoform expression (exon 2 of *KF484511* and *KF484512* isoforms) (green).

**Figure 5:**
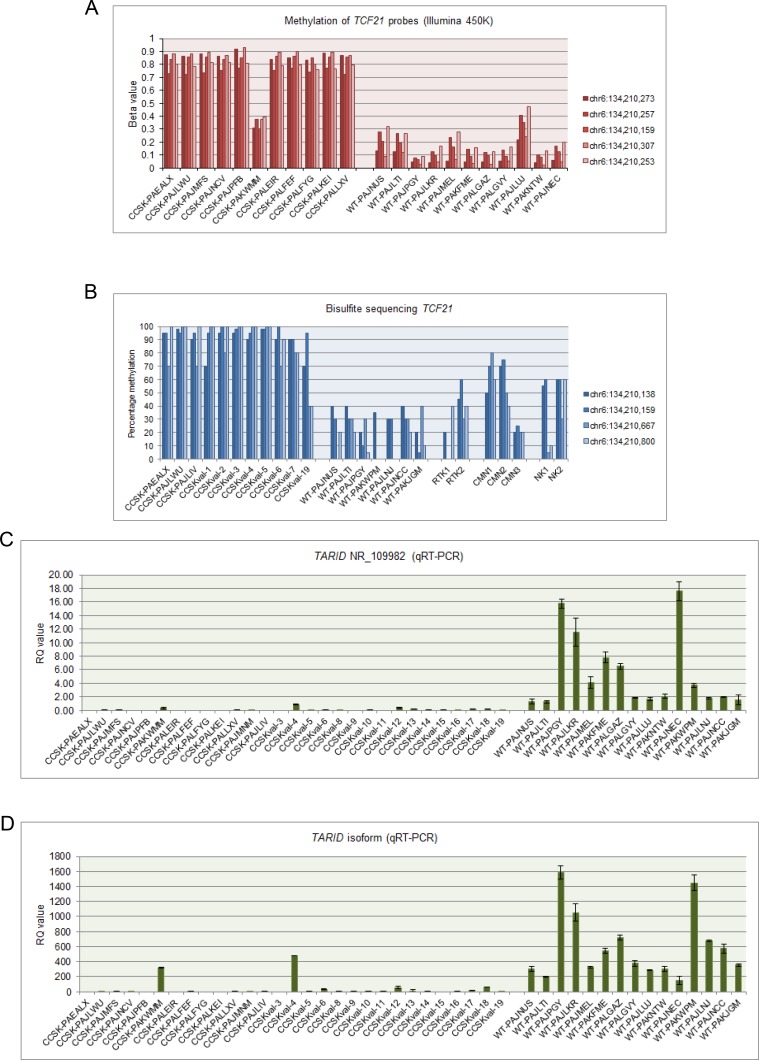
TCF21 methylation and *TARID* expression **A.** The 5 *TCF21* probes shown in Figure 4 analyzed by the 450K Illumina array in the 11 discovery set CCSKs and 11 randomly selected favorable histology Wilms tumors (WT), show high ß-values within all CCSKs except CCSK-PAKWMM, the tumor containing the t(10;17) translocation. In contrast, the favorable histology Wilms tumors show low methylation of *TCF21*. **B.** Ratio of cytosine to thymine residues following bisulfite treatment of the 4 *TCF21* locations indicated in Figure 4, show high methylation in all CCSKs (3 discovery samples, 8 validation samples), except for one validation sample (CCSK-val19) harboring the t(10;17) translocation. In contrast, the favorable histology Wilms tumor (WT), rhabdoid tumor of the kidney (RTK), congenital mesoblastic nephroma (CMN) and normal kidney (NK) samples show lower methylation of *TCF21*. **C.** *TARID* (NR_109982) expression using the primers illustrated in Figure 4, measured by quantitative RT-PCR (qRT-PCR), shows low expression of *TARID* in CCSKs (13 discovery samples, 16 validation samples) compared with favorable histology Wilms tumors (WT); in some CCSKs *TARID* was considered undetectable. **D.** *TARID* isoform expression using the primers illustrated in Figure 4, measured by qRT-PCR, likewise shows low expression in CCSKs compared with favorable histology Wilms tumors (WT).

In contrast, *TCF21*, a transcription factor involved in mesodermal development whose promoter is in a DMV, showed significant and consistent hypermethylation (average ß-value 0.78) of the promoter region in CCSKs, while the promoter region of favorable histology Wilms tumors was hypomethylated (average ß-value 0.14) (Figure 4, Figure 5A). *TCF21* promoter hypermethylation was identified in all CCSKs except the one with the t(10;17) translocation (CCSK-PAKWMM, Figure 5A). The gene expression of TCF21 was down-regulated in CCSKs in comparison with favorable histology Wilms tumors (fold change 0.05, p-value <1E-09); the CCSK with the t(10;17) translocation showed a higher level of *TCF21* expression (Figure 2).

To verify the methylation status of *TCF21*, bisulfite conversion followed by sequencing was performed on three randomly selected CCSKs from the discovery set using two primer sets, one located in the upstream promoter region and one located at the transcription start site of *TCF21* (Figure 4); two cytosine residues were analyzed per primer set, for a total of four methylation sites (Supplemental Table 3). To validate *TCF21* hypermethylation in CCSKs compared with other pediatric renal tumors, 8 CCSKs with available DNA from an independent validation set were also analyzed using the same primers; 7/8 of these CCSK samples showed higher methylation at these loci compared with 7 favorable histology Wilms tumors, 2 renal rhabdoid tumors, 3 congenital mesoblastic nephromas, and 2 normal kidney samples (Figure 5B). The CCSKs of the validation set were analyzed for the *YWHAE-NUTM2* fusion transcript, and an additional CCSK containing this translocation was identified (CCSKval-19). Of interest, similar to the translocation-bearing CCSK-PAKWMM of the discovery set, lower *TCF21* methylation levels were seen in CCSKval-19 (Figure 5B). The methylation data and fusion transcript status are provided in Supplemental Table 4.

To identify potential targets of *TCF21* within the context of the early developing kidney, and to then analyze the expression of these targets in CCSKs, we relied on the gene expression pattern of Wilms tumors, which show a gene expression pattern of the pre-induction developing kidney [24], and which do not have known genetic changes or hypermethylation of *TCF21* [25]. To accomplish this we determined the Pearson correlation coefficient (PCC) between the normalized expression of *TCF21* and all genes individually on the Affymetrix U133+2.0 array within 76 TARGET favorable histology Wilms tumors (available on the TARGET Data Matrix: http://www.target.nci.nih.gov/dataMatrix/TARGET_DataMatrix.html), and identified those genes with a PCC ≥ 0.5 or ≤ −0.5. When entering the expression of these genes in both favorable histology Wilms tumors and CCSKs into GSEA (negative and positive association separately), only those genes that positively correlated with expression were significantly differentially expressed in CCSK, and these are illustrated using hierarchical analysis in Figure 3B. This demonstrates a consistent expression pattern, with tight clustering of the CCSKs and down-regulation of the majority of the *TCF21*-associated genes.

### Analysis of *TARID* expression

*TCF21* methylation and expression has been shown to be governed by the antisense long noncoding RNA (lncRNA) *TARID*, which acts by demethylating the *TCF21* promoter [26]. The genomic structure of *TARID* according to UCSC genome browser (NR_109982) and the *TARID* isoforms previously reported by Arab *et al* are provided in Figure 4 [26]. Because *TARID* is not present within the Affymetrix 133 plus 2.0 array, we analyzed *TARID* expression by RT-qPCR in all 13 discovery CCSK samples, in 16 CCSK samples from the independent validation set with available mRNA, and in 15 randomly selected favorable histology Wilms tumors. Expression of both *TARID* (NR_109982) and the *TARID* isoforms *KF484511* and *KF484512* (isoforms reported to have the strongest demethylation activity) was much lower in CCSKs than in Wilms tumors; in most CCSKs *TARID* was undetectable (Figure 5C, 5D). A tabular representation of the *TCF21* methylation and *TARID* expression data is provided in Supplemental Table 4.

## DISCUSSION

This study comprehensively analyzes and integrates the molecular characteristics of CCSK. Our results suggest that the genome of CCSK is rather stable, similar to the genome of other pediatric tumors occurring at very young age [27]. No recurrent genetic changes were identified by copy number analysis, DNA whole genomic sequencing, or mRNA sequencing. The genetic quiescence of CCSK is supported by two recent publications. One demonstrated the absence of identifiable fusion transcripts in 19/22 CCSKs (two tumors demonstrated the *YWHAE-NUTM2* fusion transcript previously described, and a single tumor with a *TERT-IRX2* fusion transcript was identified resulting in abnormal *TERT* overexpression driven by high *IRX2* expression identified in all CCSKs) [14]. The second study analyzed 37 CCSKs for copy number changes using high-resolution genomic analysis with single nucleotide polymorphism array and demonstrated that remarkably few genetic imbalances were present in CCSKs [28]. We therefore turned to gene expression and DNA methylation for clues pointing to the pathogenesis of the majority of CCSKs.

Gene expression analysis demonstrated activation of genes involved in the Sonic Hedgehog (SHH) pathway, increased expression of *EGFR*, increased expression of *CCND1*, and increased expression of genes involved with neural development. These findings support previous publications [15, 16, 22]. SHH and increased *EGFR* expression are known to synergize and promote diverse events, including neural stem cell proliferation as well as tumor initiation and progression [29]. Therefore, it is possible that activation of these pathways within CCSKs may reflect their cell of origin rather than a tumor-initiating event [23, 30]. CCSKs are virtually exclusively found in the kidney. An origin within renal-specific progenitor cells would seem to be supported by the recent report of high expression of early renal progenitor genes *CITED1* and *FOXD1* in CCSKs [28]. However, both of these genes are also highly expressed in neuronal progenitor cells [31]. Of note, the iroquois genes (IRX genes) encode homeodomain-containing transcription factors involved in pro-neural fate [32, 33], and *YWHAE* has likewise been shown to be involved in the SHH pathway as well as neurogenesis [34-36]. We were not able to identify a gene expression pattern of early renal progenitor cells in these CCSKs, but did find evidence of increased expression of genes involved in early neural development. In summary, the available information suggests that CCSKs arise within a very specific cellular context early in renal differentiation. Normal cells within this context may have a gene expression pattern similar to early neuronal progenitor cells; alternatively an abnormal genetic or epigenetic event within this context may result in this expression pattern.

The t(10;17)(q22;p13) fusion transcript involving *YWHAE* and *NUTM2* has previously been described in 9-12% of CCSKs [12, 14]. *YWHAE* belongs to the regulatory 14-3-3 family, and plays a role in several signal transduction pathways, including Akt and Hedgehog [34, 35], as well as in neural development [36]. We identified increased expression of *YWHAE* in all CCSKs, regardless of translocation status, although the expression level of this gene was somewhat lower in the t(10;17) CCSK case. Furthermore, we demonstrate that the unique gene expression pattern previously identified in high grade endometrial stromal sarcomas harboring the t(10;17)(q22;p13) [13] is also seen in CCSKs, regardless of their translocation status. Our data therefore suggest that the underlying pathogenesis of CCSKs lacking the translocation results in a similar expression pattern.

The most striking finding identified in the current study is the presence of promoter hypermethylation and down-regulated expression of *TCF21* in all evaluated CCSK samples except those harboring the t(10;17) fusion transcript. *TCF21* (also known as *Pod-1*, *capsulin* and *epicardin*), located at 6q23, encodes a class II basic helix-loop-helix transcription factor that binds DNA and regulates differentiation and cell fate decisions during development of the heart, lung, kidney, and spleen [37]. *TCF21* is expressed in embryonic mesenchymal cells surrounding areas of epithelial development in the kidney, lung and gastrointestinal tract [38]. *TCF21* expression rapidly decreases in postnatal tissues with the exception of interstitial cells in organs including kidney, heart, lung and intestine [39]. The impact of changes in *TCF21* expression on kidney development depends on the developmental stage as well as on the particular cell being analyzed [39-41]. Gene deletion studies in chimeric mice have shown that loss of *TCF21* in the kidney prior to induction results in decreased tubulogenesis and glomerulogenesis and a failure of mesenchymal-to-epithelial transition [39-41]. Of note, suppression of *TCF21* expression by siRNA within a kidney progenitor cell line that endogenously expresses *TCF21* results in increased cell proliferation and migration and reduced smooth muscle and myofibroblast gene expression [39].

Decreased expression of *TCF21* by hypermethylation was first identified by Smith et al, who demonstrated a tumor suppressor function in head and neck squamous cell carcinoma and non-small-cell lung cancer [42]. Since then, *TCF21* promoter hypermethylation has been associated with poor outcome in various tumor types, including metastatic melanoma, lung adenocarcinomas, squamous cell lung cancers, clear cell renal cell carcinoma and other urological cancers [43-47]. Many transcription factors and gene regulators responsible for early development have been shown to have promoters within DNA methylation valleys (DMVs); such genes later in life frequently gain abnormal methylation in cancer [23]. Of note, the promoter of *TCF21* is within a DMV [23].

Recently Arab *et al* provided evidence that the antisense long noncoding RNA (lncRNA) *TARID* activates *TCF21* expression by inducing promoter demethylation [26]. They demonstrate that *TARID* accomplishes this by interacting with both the *TCF21* promoter and with *GADD45A*, an adaptor protein that tethers the nucleotide excision repair and demethylation machineries to sites of DNA demethylation. We show that CCSKs have a significantly lower *TARID* expression compared to Wilms tumors, negatively correlating with the level of *TCF21* promoter methylation. This suggests the possibility that the direct cause of *TCF21* hypermethylation in CCSKs may be decreased *TARID* expression, although we have not identified a genetic cause for this decreased expression (eg mutation or copy number loss). *TCF21* has also been shown to be a target of the polycomb group repressor *EZH2*, whose function is to establish the H3K27me3 histone mark, and *TCF21* is significantly up-regulated following silencing of *EZH2* [48], suggesting that histone modifications may ultimately regulate *TCF21* expression, perhaps through *TARID* expression.

In summary hypermethylation and decreased expression of a tumor suppressor gene known to be active in renal development supports the hypothesis that epigenetic regulation of *TCF21* very early in renal development may be involved in the pathogenesis of CCSKs. Future studies are needed to functionally verify the role of *TCF21* down-regulation, to identify the proximate cause of its down-regulation, and to tie this to the unique expression patterns of CCSKs. If hypermethylation of *TCF21* is involved in the pathogenesis, this may provide a rationale for treatment of patients with CCSK with demethylating agents. In lung cancer cell lines and head and neck squamous cell carcinoma cell lines, *TCF21* expression could be restored through treatment with decitabine, one of the clinically available demethylating agents [42].

## MATERIALS AND METHODS

This study is part of the “Therapeutically Applicable Research to Generate Effective Treatments” (TARGET) initiative which provides access to the gene expression, chromosome copy number and methylation data (raw, normalized, and level 3 data), as well as the results of sequence analysis (i.e. MAF and summary files), detailed methods and clinical information through the TARGET Data Matrix (http://target.nci.nih.gov/dataMatrix/TARGET_DataMatrix.html). The data provided are fully annotated within MIAME compliant MAGE-TAB files describing the methods, the specimen processing details and the quality control parameters for each platform. The aligned sequencing data (BAM and FASTQ files) are deposited in the Sequence Read Archive (SRA) at the National Center for Biotechnology Information, and are accessible through the database of genotypes and phenotypes (dbGAP, http://www.ncbi.nlm.nih.gov/gap) under the accession number phs000466. A summary of the methods used in this study is provided below.

### Sample selection and preparation

The primary goal of this study is to identify recurrent pathogenetic changes that result in the development of CCSK. A secondary goal is to identify genetic changes associated with relapse. CCSKs are quite rare, and adequate tumor/normal sample pairs sufficient for this project are limited. To address our goals given this limitation, from the 110 CCSKs registered on National Wilms Tumor Study-5 (NWTS-5) we identified those with sufficient frozen primary tumor material and blood or normal kidney tissue. From these, seven cases were identified as high risk because they relapsed. We then identified an additional seven CCSKs who did not relapse, selecting those with the highest available stage in order to maximize our ability to detect high risk features. Thirteen of the 14 selected cases passed the quality tests following analyte extraction and these represent the discovery set. The clinical features of the discovery set are provided in Supplemental Table 4. The six patients who relapsed (one of the relapsed cases failed the quality tests) did so 229-712 days following diagnosis (mean 499 days); the seven patients who did not relapse were followed from 244-4440 days (mean 2235 days).

To validate the findings, an independent validation set was identified from a total of 19 additional CCSK cases; many available samples included only DNA or RNA. The majority of the validation cases were drawn from the remaining NWTS-5 CCSKs; four cases were not registered on clinical protocols and were obtained from the Children's Oncology Group (COG) Biopathology Center tumor bank. The clinical features available for the validation set are likewise provided in Supplemental Table 4.

For all samples, frozen tissue was obtained from the COG Biopathology Center. The pathologic diagnosis was provided by central pathology review. Studies were performed with the approval of the Lurie Children's Hospital Institutional Review Board. All tumors had > 80% tumor cellularity determined by frozen section analysis of the same tissue sample that underwent extraction for nucleotide analysis.

### Chromosome segment copy number analysis

Nucleic acid labeling, hybridization, and array scanning were performed on 11 CCSKs according to the manufacturer's protocol for the Affymetrix 6.0 SNP array (Affymetrix, Santa Clara, CA, USA) and processed with the Affymetrix Genotyping Console (GTC) 4.0 software. Reference normalization was performed as described by Pounds et al [49]. Circular binary segmentation (CBS) was performed using DNAcopy from BioConductor (http://www.bioconductor.org). Segmented regions of autosomal chromosomes containing at least 8 markers in which the log2 value was > +0.5 or < −0.5 were considered regions of gain or loss, respectively. For the other 2 CCSK samples, copy number was assessed by using relative coverage generated by whole genome sequencing.

### Whole genome sequencing

Whole genome sequencing was done by Complete Genomics (CGI, Mountain View, CA, USA) [50]. Alignment of reads to the NCBI Build 37 reference human genome assembly and mutations “calling” was performed by the CGI Cancer Sequencing service, as described in the TARGET Data Matrix. Information regarding the transcript and protein effects of the variants, as well as the presence of the variants in a number of different lists, including the Catalogue of Somatic Mutations in Cancer (COSMIC) and The Cancer Genome Atlas (TCGA), is obtained using Oncotator (http://www.broadinstitute.org/oncotator). Variants that passed filtering parameters (somatic score ≥ −10, somatic rank ≥ 0.1 and Fisher's Exact Test [FET] score ≥ 13) were additionally analyzed by MutSig [51], which takes as input the number of bases successfully sequenced for each gene, the number of observed mutations per gene and the empirically derived background mutation rate, and applies a standard binomial test to determine if the number of observed mutations per gene is greater than expected by chance. Given the low mutation rate in CCSK, this variant list was combined with the variants from the TARGET favorable histology Wilms tumors available at the time (n =76) for MutSig analysis.

### mRNA sequencing

TruSeq stranded total RNA kits were used to construct total RNA libraries according to the manufacturer's protocol (Illumina, San Diego, CA, USA). The reads were mapped against NCI Build 37/hg19 human reference genome by using TopHat2 with fusion parameters. DeFuse and TopHat2 fusion were used to predict the presence of fusion proteins. SAMtools mpileup was used to count the number of reads uniquely mapped to a particular position identified as variant by whole genome sequencing in the same sample. If there were reads supporting a variant base in RNASeq, the total reads were counted and Variant Allele Frequency (VAF) was calculated.

### Gene expression

Total RNA was used for gene expression analysis using the Affymetrix 133 plus 2.0 array (Affymetrix, Santa Clara, CA, USA), performed according to the manufacturer's protocol. The arrays were analyzed using Gene-Chip Operating Software (GCOS) and Robust Multichip Average (RMA) normalization was performed. Differentially expressed genes were identified using a significance analysis of microarrays (SAM) [52]; q-values of < 0.01 and fold changes of > 2 were considered significant. Gene Set Enrichment Analysis (GSEA), version 2.0.14, (http://www.broadinstitute.org/gsea) [53] was performed using 1000 permutations and phenotype permutation. Lists with at least 50 genes of canonical pathways, biologic processes and oncogenic signatures with a false discovery rates (FDR) of < 20% and p-value of < 0.05 were considered significant. Pearson correlation coefficient (PCC) calculation was performed using the RMA-normalized Level 3 gene expression data for 76 favorable histology Wilms tumors available in the TARGET Data Matrix. Hierarchical clustering was performed by using GenePattern's HierarchicalClustering module (column distance measure = Pearson correlation; row distance measure = Pearson correlation; clustering method = pairwise average-linkage) and were visualized by the HierarchicalClusteringViewer module.

### Global DNA methylation analysis

Methylation analysis was performed on 11 samples for which sufficient DNA was available, using Illumina Infinium Human Methylation 450K BeadChips (Illumina, San Diego, CA, USA) according to the manufacturer's protocol [54]. ß-values were calculated from GenomeStudio v2010.1. Integrative genome viewer (IGV) was utilized to visualize methylation data (http://www.broadinstitute.org/igv/). Data were correlated with gene expression data as follows: methylation probes located in the gene body, or within 10k base pairs upstream or downstream of a gene were identified. For each gene, the expression was determined by using the probe with the highest expression. For each probe and gene pair, the correlation between methylation and gene expression was analyzed using the Generalized Linear Model (GLM), which is implemented in R (http://www.R-project.org/). P-values were adjusted for multiple comparisons using the multitest package in R. A negative t-value indicates that the methylation and expression levels are inversely correlated. A correlation with adjusted p-value < 0.05 was considered significant.

### DNA methylation analysis of *TCF21* following bisulfite conversion

To verify and validate the array methylation results of *TCF21*, real-time quantitative methylation-specific polymerase chain reaction was performed with primers and probes to the promoter and transcription start site specifically designed to bind to bisulfite-converted DNA, as previously described by Costa et al [44]. Primer and probe sequences are provided in Supplemental Table 3. The methyl cytosine of each CpG site was quantified by using Chromas Lite (Technelysium, South Brisbane, QLD, Australia) to compare the peak height of the cytosine signal with the sum of the cytosine and thymine peak height signals. CpG sites with ratio ranges 0–0.20, 0.21–0.80, and 0.81–1.0 were considered unmethylated, partially methylated, and fully methylated, respectively.

### Real time quantitative polymerase chain reaction (RT-qPCR)

Relative expression of the long noncoding RNA *TARID* was determined by RT-qPCR. Gene-specific primers and probe were generated by Life Technologies for a Custom TaqMan Gene Expression Assay Kit by using the *TARID* sequence ID (NR_109982) and described *TARID* isoforms (Supplemental Table 3) [26]. cDNA was generated from 50 ng of total RNA using the Applied Biosystems High Capacity cDNA RT kit. Values were normalized to the housekeeping gene GAPDH. Data are presented as the relative quantitation (RQ) value, which was calculated by using a single low-expression sample as the calibrator.

### *YHWAE-NUTM2* translocation analysis

RT-PCR analysis was performed using previously reported primers for *YHWAE* exon 5 and *NUTM2* exon 2 [12]. Briefly, 50 ng of DNase-treated RNA was used for the reverse transcription reaction using the High-Capacity cDNA Reverse Transcription Kit (Life Technologies, Grand Island, NY), according to the manufacturer's protocol. PCR was performed by using the Invitrogen Platinum Taq DNA Polymerase (Invitrogen/Life Technologies).

## SUPPLEMENTARY MATERIAL TABLES


