# Supplementary material for: TCF21 hypermethylation in genetically quiescent clear cell sarcoma of the kidney
Source: Oncotarget. 2015 Jun 28;6(18):15828–41. doi: 10.18632/oncotarget.4682 (PMC4599240; doi:10.18632/oncotarget.4682)
Supplement: Supplementary file 1 [file oncotarget-06-15828-s001.pdf]

TCF21 hypermethylation in genetically quiescent clear cell sarcoma of the kidney

Supplementary Material

Supplemental Table 1. Verified somatic and germline variants

Chr	Position (GRCh37)	Ref	Alt	TARGET_CASE_ID	Hugo_Symbol	Variant_Classification	Variant Type	Mutation_Status	Tumor_ReadCount_Alt	Tumor_ReadCount_Ref	Normal_ReadCount_Alt	Normal_ReadCount_Ref	Cosmic_Gene	Entrez_Gene_id	RNAseq.TotCov	RNAseq VAF	MutSig p-value	Amino acid change	PPH2_Class	PPH2_Prob	PPH2_FDR
9	138706970	G	C	PAJNCV	CAMSAP1	MISSENSE	SNP	Somatic	20	7	0	34	Yes	157922	177	0.51	0.40	K/N(AAG/AAC)	deleterious	1	0
10	12143052	C	G	PALFEF	DHTKD1	MISSENSE	SNP	Somatic	20	26	0	42	Yes	55526	37	0.41	0.32	R/G(CGT/GGT)	deleterious	1	0
14	104641803	C	T	PAEALX	KIF26A	MISSENSE	SNP	Somatic	13	23	0	33	Yes	26153	129	0.33	0.77	P/L(CCG/CTG)	deleterious	0.768	0.199
22	22318567	C	T	PAKWMM	TOP3B	MISSENSE	SNP	Somatic	8	17	0	24	Yes	8940	52	0.38	0.72	Q/R(CAG/CGG)	neutral	0.029	0.328
5	108714626	C	T	PAJLWU	PJA2	MISSENSE	SNP	Somatic	21	18	0	33	Yes	9867	515	0.45	0.26	M/V(ATG/GTG)	neutral	0.002	0.556
2	73675690	A	G	PALLXV	ALMS1	MISSENSE	SNP	Germline	35	32	24	32	Yes	7840	168	0.61	0.60	Y/C(TAT/TGT)	deleterious	0.992	0.12
2	73827844	G	A	PAJPFB	ALMS1	MISSENSE	SNP	Germline	25	21	28	34	Yes	7840	144	0.47	0.60	R/Q(CGA/CAA)	deleterious	0.695	0.207
6	38562038	G	A	PAEALX	BTBD9	MISSENSE	SNP	Germline	16	20	20	14	Yes	114781	30	0.47	0.16	I/T(ATT/ACT)	deleterious	0.605	0.218
6	38561864	A	G	PAJPFB	BTBD9	MISSENSE	SNP	Germline	22	25	15	21	Yes	114781	40	0.30	0.16	A/V(GCC/GTC)	deleterious	0.793	0.197
11	65351829	G	C	PAJMFS	EHBP1L1	MISSENSE	SNP	Germline	29	21	18	20	Yes	254102	85	0.54	0.45	A/P(GCC/CCC)	deleterious	0.889	0.182
11	65347830	G	A	PAJNCV	EHBP1L1	MISSENSE	SNP	Germline	24	38	38	34	Yes	254102	90	0.50	0.45	A/T(GCA/ACA)	deleterious	0.96	0.157
7	5410697	C	G	PAJLIV	TNRC18	MISSENSE	SNP	Germline	26	20	20	20	Yes	84629	51	0.57	0.47	D/E(GAC/GAG)	deleterious	0.683	0.209
7	5396875	G	T	PALFEF	TNRC18	MISSENSE	SNP	Germline	24	23	25	19	Yes	84629	509	0.46	0.47	E/D(GAA/GAC)	deleterious	0.956	0.16
1	19479968	G	A	PAJNCV	UBR4	MISSENSE	SNP	Germline	11	21	18	15	Yes	23352	235	0.50	0.25	M/T(ATG/ACG)	deleterious	0.997	0.0665
1	19500004	C	T	PALLXV	UBR4	MISSENSE	SNP	Germline	24	29	31	36	Yes	23352	275	0.44	0.25	K/E(AAA/GAA)	deleterious	0.906	0.179
1	19480266	G	A	PALEIR	UBR4	MISSENSE	SNP	Germline	30	29	30	22	Yes	23352	253	0.37	0.25	V/A(GTG/GCG)	deleterious	0.891	0.182

Supplemental Table 2. Probes showing a significant correlation between expression and methylation ($p < 0.05$) selected for further analysis after filtering

Gene	Probe	T. value	P. value	FDR	Chr	Start Position (GRCh37)	UCSC refgene group	Relation to UCSC CpG island	UCSC CpG island name	Ave. beta-value CCSK	Stand. Dev. CCSK	Ave. beta-value WT	Stand. Dev. WT	T-test beta-values CCSK vs WT	Differential expression	Promoter located in DMV
EMX2	p222156	21.75	4.3E-09	7.3E-06	10	119284710	Body	Island	10:119293917-119297675	0.087	0.10	0.173	0.14	0.00074	Down in CCSK	Yes
EMX2	p85514	10.26	7.0E-06	3.7E-03	10	119292300	Body	N_Shore	10:119293917-119297675	0.160	0.18	0.149	0.15	.	Down in CCSK	.
EMX2	p176416	19.03	1.4E-08	1.9E-05	10	119293967	Body	Island	10:119293917-119297675	0.085	0.16	0.127	0.15	.	Down in CCSK	.
EMX2	p295836	9.42	5.9E-06	3.4E-03	10	119294173	Body	Island	10:119293917-119297675	0.175	0.18	0.212	0.22	.	Down in CCSK	.
EMX2	p420696	32.97	1.1E-10	4.0E-07	10	119295056	Body	Island	10:119293917-119297675	0.111	0.21	0.145	0.17	.	Down in CCSK	.
EMX2	p337229	15.73	7.5E-08	7.5E-05	10	119295770	Body	Island	10:119293917-119297675	0.189	0.18	0.634	0.24	.	Down in CCSK	.
EMX2	p209891	12.63	5.0E-07	4.0E-04	10	119295875	Body	Island	10:119293917-119297675	0.176	0.16	0.589	0.23	.	Down in CCSK	.
EMX2	p152447	24.42	1.5E-09	3.3E-06	10	119296182	Body	Island	10:119293917-119297675	0.187	0.14	0.538	0.22	.	Down in CCSK	.
EMX2	p372292	25.63	5.3E-11	3.0E-07	10	119296297	Body	Island	10:119293917-119297675	0.105	0.16	0.430	0.21	.	Down in CCSK	.
EMX2	p120750	20.48	7.4E-09	1.2E-05	10	119296305	Body	Island	10:119293917-119297675	0.161	0.18	0.599	0.23	.	Down in CCSK	.
EMX2	p7949	10.73	2.0E-06	1.3E-03	10	119296942	Body	Island	10:119293917-119297675	0.130	0.18	0.172	0.08	.	Down in CCSK	.
EMX2	p93101	17.08	3.6E-08	4.0E-05	10	119297439	Body	Island	10:119293917-119297675	0.180	0.18	0.074	0.02	.	Down in CCSK	.
EMX2	p363531	10.86	1.8E-06	1.3E-03	10	119297575	Body	Island	10:119293917-119297675	0.136	0.14	0.048	0.01	.	Down in CCSK	.
EMX2	p344900	26.65	7.1E-10	2.0E-06	10	119311636	Unknown	Island	10:119311204-119312104	0.143	0.20	0.654	0.30	.	Down in CCSK	.
EMX2	p179228	15.52	8.4E-08	8.1E-05	10	119312077	Unknown	Island	10:119311204-119312104	0.249	0.20	0.601	0.37	.	Down in CCSK	.
EMX2	p159648	19.13	1.3E-08	1.9E-05	10	119312691	Unknown	N_Shore	10:119312766-119313563	0.105	0.12	0.408	0.21	.	Down in CCSK	.
EMX2	p286454	8.90	9.3E-06	4.6E-03	10	119312767	Unknown	Island	10:119312766-119313563	0.137	0.16	0.410	0.30	.	Down in CCSK	.
EMX2	p85083	16.99	3.8E-08	4.0E-05	10	119312950	Unknown	Island	10:119312766-119313563	0.144	0.12	0.286	0.24	.	Down in CCSK	.
EMX2	p305305	7.73	2.9E-05	1.2E-02	10	119313075	Unknown	Island	10:119312766-119313563	0.210	0.16	0.338	0.32	.	Down in CCSK	.
HOXA1	p172458	-9.64	4.8E-06	2.2E-02	7	27133106	3'UTR	N_Shore	7:27134097-27134303	0.804	0.20	0.682	0.19	0.00917	NS	Yes
HOXA1	p419577	-22.46	3.3E-09	6.7E-05	7	27134109	3'UTR;Body	Island	7:27134097-27134303	0.833	0.24	0.650	0.26	.	NS	.
HOXA1	p149265	-14.43	1.6E-07	1.3E-03	7	27134225	3'UTR;Body	Island	7:27134097-27134303	0.733	0.23	0.494	0.23	.	NS	.
HOXA1	p341125	-16.19	5.8E-08	6.0E-04	7	27134259	3'UTR;Body	Island	7:27134097-27134303	0.725	0.23	0.370	0.20	.	NS	.
HOXA1	p392703	-23.66	2.1E-09	6.7E-05	7	27134369	3'UTR;Body	N_Shore	7:27135342-27136736	0.787	0.24	0.643	0.26	.	NS	.
HOXA1	p332067	-10.28	2.8E-06	1.5E-02	7	27244370	Unknown	N_Shore	7:27244530-27245297	0.722	0.12	0.318	0.16	.	NS	.
HOXA1	p43423	-8.06	2.1E-05	4.9E-02	7	27244564	Unknown	Island	7:27244530-27245297	0.814	0.19	0.057	0.02	.	NS	.
IRX4	p140188	13.60	2.6E-07	2.1E-03	5	1878205	Body	Island	5:1874907-1879032	0.156	0.16	0.070	0.08	0.04953	NS	Yes
IRX4	p83993	14.51	1.5E-07	1.5E-03	5	1878499	Body	Island	5:1874907-1879032	0.231	0.14	0.103	0.13	.	NS	.
IRX4	p330815	10.95	1.7E-06	8.2E-03	5	1878672	Body	Island	5:1874907-1879032	0.212	0.19	0.085	0.11	.	NS	.
IRX4	p45670	8.63	1.2E-05	2.8E-02	5	1880310	Body	N_Shore	5:1881924-1887743	0.171	0.17	0.128	0.07	.	NS	.
IRX4	p117113	17.05	3.7E-08	7.3E-04	5	1884212	TSS1500	Island	5:1881924-1887743	0.077	0.04	0.063	0.01	.	NS	.
IRX4	p275677	8.08	2.0E-05	3.4E-02	5	1937365	TSS1500	Island	5:1881924-1887743	0.083	0.04	0.073	0.01	.	NS	.
MCF2L	p57183	7.06	5.9E-05	4.9E-02	13	113698416	Body	N_Shelf	13:113701833-113702280	0.146	0.07	0.635	0.15	0.00326	Down in CCSK	No
MCF2L	p299101	6.98	6.4E-05	4.9E-02	13	113698431	Body	N_Shelf	13:113701833-113702280	0.075	0.04	0.503	0.16	.	Down in CCSK	.

MCF2L	p261779	8.27	1.7E-05	2.1E-02	13	113698621	Body	N_Shelf	13:113701833-113702280	0.110	0.09	0.512	0.15	.	Down in CCSK	.
MCF2L	p205523	9.80	4.2E-06	9.5E-03	13	113698688	Body	N_Shelf	13:113701833-113702280	0.076	0.04	0.298	0.09	.	Down in CCSK	.
MCF2L	p14287	7.19	5.1E-05	4.9E-02	13	113698994	Body	N_Shelf	13:113701833-113702280	0.127	0.09	0.716	0.19	.	Down in CCSK	.
OSR2	p29251	-8.15	1.9E-05	2.9E-02	8	99951151	Unknown	N_Shore	8:99952020-99954686	0.773	0.20	0.536	0.21	0.02043	Up in CCSK	Yes
OSR2	p136556	-9.23	6.9E-06	2.4E-02	8	99952047	Unknown	Island	8:99952020-99954686	0.825	0.20	0.070	0.08	.	Up in CCSK	.
OSR2	p463677	-8.93	9.1E-06	2.4E-02	8	99960500	Unknown	Island	8:99960497-99961438	0.778	0.22	0.039	0.01	.	Up in CCSK	.
OSR2	p32753	-7.99	2.2E-05	3.0E-02	8	99961874	Body	S_Shore	8:99960497-99961438	0.762	0.22	0.301	0.23	.	Up in CCSK	.
OSR2	p340044	-7.37	4.2E-05	4.1E-02	8	99962347	Body	S_Shore	8:99960497-99961438	0.787	0.21	0.670	0.21	.	Up in CCSK	.
PAX2	p56440	39.79	2.0E-11	1.4E-07	10	102586454	Body	Island	10:102586125-102588109	0.172	0.22	0.230	0.15	0.00013	Down in CCSK	Yes
PAX2	p446648	10.58	2.2E-06	1.5E-03	10	102586999	3'UTR;Body	Island	10:102586125-102588109	0.133	0.12	0.270	0.12	.	Down in CCSK	.
PAX2	p98642	25.40	1.1E-09	2.5E-06	10	102587322	3'UTR;Body	Island	10:102586125-102588109	0.158	0.16	0.354	0.18	.	Down in CCSK	.
PAX2	p409027	45.15	6.4E-12	7.2E-08	10	102587844	3’UTR	Island	10:102586125-102588109	0.224	0.22	0.566	0.23	.	Down in CCSK	.
PAX2	p319793	8.47	1.4E-05	6.7E-03	10	102588959	3’UTR	N_Shore	10:102590122-102590402	0.226	0.18	0.519	0.26	.	Down in CCSK	.
PAX2	p274928	19.32	1.2E-08	1.8E-05	10	102589102	3’UTR	N_Shore	10:102590122-102590402	0.213	0.24	0.507	0.33	.	Down in CCSK	.
PAX2	p159492	22.63	3.0E-09	5.7E-06	10	102589250	3’UTR	N_Shore	10:102590122-102590402	0.178	0.22	0.489	0.31	.	Down in CCSK	.
PAX2	p336155	6.99	6.4E-05	2.2E-02	10	102589532	3’UTR	N_Shore	10:102590122-102590402	0.247	0.17	0.588	0.24	.	Down in CCSK	.
SOX1	p44198	15.24	9.8E-08	3.7E-04	13	112715454	Unknown	Island	13:112715359-112716234	0.084	0.08	0.042	0.01	0.02985	Up in CCSK	Yes
SOX1	p158252	9.53	5.3E-06	1.0E-02	13	112717207	Unknown	Island	13:112717125-112717421	0.163	0.09	0.055	0.01	.	Up in CCSK	.
SOX1	p181584	7.00	6.3E-05	4.9E-02	13	112717244	Unknown	Island	13:112717125-112717421	0.141	0.15	0.019	0.01	.	Up in CCSK	.
SOX1	p335152	8.64	1.2E-05	1.7E-02	13	112717484	Unknown	N_Shore	13:112715359-112716234	0.154	0.14	0.054	0.01	.	Up in CCSK	.
SOX1	p220516	20.37	7.7E-09	8.7E-05	13	112728799	Unknown	N_Shore	13:112726281-112728419	0.067	0.08	0.112	0.09	.	Up in CCSK	.
TCF21	p375100	-54.27	1.2E-12	1.5E-07	6	134210159	TSS200	N_Shore	6:134210639-134211218	0.802	0.16	0.208	0.17	0.00000	Down in CCSK	Yes
TCF21	p230346	-18.82	1.6E-08	6.7E-04	6	134210253	TSS200	N_Shore	6:134210639-134211218	0.757	0.12	0.305	0.23	.	Down in CCSK	.
TCF21	p191762	-17.67	2.7E-08	6.7E-04	6	134210257	TSS200	N_Shore	6:134210639-134211218	0.712	0.11	0.268	0.18	.	Down in CCSK	.
TCF21	p232130	-18.62	1.7E-08	6.7E-04	6	134210273	Exon1;5'UTR	N_Shore	6:134210639-134211218	0.817	0.16	0.139	0.12	.	Down in CCSK	.
TCF21	p424712	-13.74	2.4E-07	3.3E-03	6	134210307	Exon1;5'UTR	N_Shore	6:134210639-134211218	0.835	0.15	0.169	0.20	.	Down in CCSK	.

Supplemental Table 3. Primer sequences

I. Bisulfite sequencing (*TCF21*)

Primer set	Forward primer	Reverse primer
TCF21 (Costa et al) *	AGGATTTTTAAGAGGTGG	AAACCTTACTCAACACTC
TCF21 (Zymo) **	AAGAGAGAAAGAAGGTTTTTTATAGATGGATAG	ACTCTCTATTTCCCCACTCCCAAAATC

* Costa et al. Epigenetics 2011;6(9):1120-1130
** http://www.zymoresearch.com/tools/bisulfite-primer-seeker

II. RT-qPCR primers (*TARID*)

Primer set	Forward primer	Reverse primer
TARID (Life Technologies) ***	CGACCAACTTCCTCTACTGCTT	TGTATAGTTGAAGTGACCTCCCAAGA
TARID (Life Technologies) ****	CATGTACTTACCAGCCACCTTCTC	AGATGGACAGAACATGCTGCTT

*** Life Technologies Catalog #4331348, Assay ID AI89LFR, sequence ID against which primers/probes were generated: NR_109982
**** Life Technologies Catalog #4331348, Assay ID AII1N1E, sequence ID against which primers/probes were generated: KF484511 and KF484512

Supplemental Table 4. Clinical features, *TCF21* methylation, *TARID* expression, translocation analysis

I. Discovery set

Sample Designation	Gender	Age (months)	Stage	Relapse	Event Free Survival (days)	Vital Status	*TCF21* Illumina 450K beta value, average (Fig 5A)	*TCF21* bisulfite sequencing, average methylation level (Fig 5B)	*TARID* NR_109982 expression, RQ value (Fig 5C)	*TARID* isoform expression, RQ value (Fig 5D)	Translocation Analysis
CCSK-PAJLWU	Male	19	III	None	4440	Alive	0.821	98.3%	0.044	0.75	Negative
CCSK-PAEALX	Male	8	III	None	3058	Alive	0.825	90.0%	Undetectable	Undetectable	Negative
CCSK-PAJMFS	Male	32	III	None	1626	Alive	0.836	NA	0.018	2.65	Negative
CCSK-PAJNCV	Male	37	III	None	2972	Alive	0.827	NA	Undetectable	1.69	Negative
CCSK-PAJPFB	Male	17	III	None	1966	Alive	0.854	NA	Undetectable	Undetectable	Negative
CCSK-PAKWMM	Male	8	III	Relapse	712	Dead	0.350	NA	0.273	318.51	Positive
CCSK-PALEIR	Male	15	II	Relapse	595	Alive	0.828	NA	Undetectable	Undetectable	Negative
CCSK-PALFEF	Male	20	II	None	244	Alive	0.836	NA	Undetectable	2.15	Negative
CCSK-PALFYG	Male	9	II	Relapse	490	Dead	0.798	NA	Undetectable	Undetectable	Negative
CCSK-PALKEI	Male	35	III	None	1339	Alive	0.834	NA	Undetectable	Undetectable	Negative
CCSK-PALLXV	Male	15	III	Relapse	468	Dead	0.823	NA	0.030	2.36	Negative
CCSK-PAJMNM	Male	16	II	Relapse	500	Alive	NA	NA	0.009	0.22	Negative
CCSK-PAJLIV	Male	8	III/IV	Relapse	229	Dead	NA	88.8%	Undetectable	1.34	Negative

II. Validation set

CCSKval-1	Male	26	I	NA	NA	NA	NA	91.3%	NA	NA	Negative
CCSKval-2	Male	10	II	NA	NA	NA	NA	93.8%	NA	NA	NA
CCSKval-3	Female	71	II	NA	NA	NA	NA	98.3%	Undetectable	Undetectable	Negative
CCSKval-4	Male	38	NA	NA	NA	NA	NA	96.3%	0.858	481.02	Negative
CCSKval-5	Male	14	NA	NA	NA	NA	NA	99.0%	0.006	3.25	Negative
CCSKval-6	Female	9	NA	NA	NA	NA	NA	87.5%	0.098	34.84	Negative
CCSKval-7	NA	NA	NA	NA	NA	NA	NA	85.0%	NA	NA	NA
CCSKval-8	Male	24	NA	NA	NA	NA	NA	No	0.006	8.25	Negative
CCSKval-9	Male	102	III	NA	NA	NA	NA	No	Undetectable	2.12	Negative
CCSKval-10	Female	22	III	NA	NA	NA	NA	No	0.051	1.24	NA
CCSKval-11	Male	44	III	None	NA	NA	NA	No	Undetectable	4.89	Negative
CCSKval-12	Female	49	III	NA	NA	NA	NA	No	0.419	55.04	Negative
CCSKval-13	Male	50	III	NA	NA	NA	NA	No	0.188	12.21	Negative
CCSKval-14	Female	63	III	NA	NA	NA	NA	No	0.034	3.93	Negative
CCSKval-15	Male	10	II	NA	NA	NA	NA	No	0.056	Undetectable	Negative
CCSKval-16	Male	45	IV	Relapse	NA	NA	NA	No	0.008	1.90	Negative
CCSKval-17	Male	8	III	NA	NA	NA	NA	No	0.242	18.06	Negative
CCSKval-18	Female	12	III	None	NA	NA	NA	No	0.188	61.24	Negative
CCSKval-19	Male	9	II	Relapse	NA	NA	NA	61.3%	0.006	0.50	Positive

III. Wilms tumor validation set

WT-PAJNUS	Female	29	III/IV	Relapse	203	Dead	0.206	22.5%	1.281	300.18	NA
WT-PAJLTI	Male	31	I	Relapse	180	Alive	0.194	30.0%	1.265	200.26	NA
WT-PAJPGY	Female	70	II	Relapse	432	Alive	0.062	16.3%	15.746	1590.50	NA
WT-PAJLKR	Female	74	III/IV	Relapse	150	Alive	0.096	NA	11.512	1054.57	NA
WT-PAJMEL	Female	44	III/IV	Relapse	373	Alive	0.160	NA	4.102	327.06	NA
WT-PAKFME	Female	56	III	Relapse	281	Alive	0.094	NA	7.819	548.29	NA
WT-PALGAZ	Female	7	I	Relapse	152	Dead	0.084	NA	6.495	719.18	NA
WT-PALGVY	Female	39	III	Relapse	370	Alive	0.099	NA	1.862	375.56	NA
WT-PAJLUJ	Female	76	IIIB/IV	Relapse	250	Alive	0.339	NA	1.684	285.45	NA
WT-PAKNTW	Male	157	III	Relapse	290	Alive	0.075	NA	2.089	306.82	NA
WT-PAJNEC	Female	48	III	Relapse	394	Dead	0.121	NA	17.590	153.95	NA
WT-PAKWPM	Male	63	II	Relapse	179	Alive	NA	8.8%	3.734	1449.00	NA
WT-PAJLNJ	Female	11	II	Relapse	250	Dead	NA	15.0%	1.783	675.21	NA
WT-PAJNCC	Male	26	II	Relapse	427	Alive	NA	30.0%	1.990	572.72	NA
WT-PAKJGM	Male	33	III/IV	Relapse	201	Dead	NA	18.8%	1.517	355.64	NA
